# Supplementary material for: MetaRibo-Seq measures translation in microbiomes
Source: Nat Commun. 2020 Jun 29;11:3268. doi: 10.1038/s41467-020-17081-z (PMC7324362; doi:10.1038/s41467-020-17081-z)
Supplement: Supplementary file 10 — Supplementary Data 7 [file 41467_2020_17081_MOESM10_ESM.zip › File2/Confidence_High_Taxonomy/155255_out.krona.html]

Javascript must be enabled to view this page.

members
magnitude
magnitudeUnassigned
count
unassigned
taxon
rank

155255\_out

272

272
2
superkingdom

976
1
phylum

1
200643
class

171549
1
order

family
1
815

816
1
genus

species

SRS015960\_contig\_number\_contig-100\_555.111794
46506
1

1239
271
phylum

186801
271
class

order

SRS013215\_contig\_number\_contig-100\_3174.3174SRS014613\_contig\_number\_contig-100\_7049.94316SRS015190\_contig\_number\_7755SRS015782\_contig\_number\_contig-100\_6446.201192SRS016335\_contig\_number\_contig-100\_3985.210806SRS018575\_contig\_number\_contig-100\_2403.31426SRS104485\_contig\_number\_contig-100\_88.43893
271
7
186802

114
216572
family

1
1952972

SRS1041140\_contig\_number\_19208
species

459786
113
genus

species
108

SRS011084\_contig\_number\_contig-100\_2000.198037SRS011405\_contig\_number\_13453SRS013951\_contig\_number\_7875SRS014415\_contig\_number\_contig-100\_826.27679SRS014948\_contig\_number\_contig-100\_698.140992SRS014979\_contig\_number\_30119SRS015264\_contig\_number\_20159SRS015579\_contig\_number\_23717SRS015590\_contig\_number\_6168SRS015794\_contig\_number\_12625SRS016018\_contig\_number\_contig-100\_386.103400SRS016753\_contig\_number\_3008SRS016954\_contig\_number\_18863SRS017103\_contig\_number\_28950SRS017191\_contig\_number\_14496SRS017307\_contig\_number\_30684SRS017521\_contig\_number\_contig-100\_4815.231128SRS017821\_contig\_number\_33141SRS018133\_contig\_number\_12224SRS018351\_contig\_number\_17061SRS018623\_contig\_number\_20075SRS018817\_contig\_number\_18398SRS019030\_contig\_number\_13327SRS019685\_contig\_number\_contig-100\_3246.197780SRS019693\_contig\_number\_8873SRS019910\_contig\_number\_24894SRS020328\_contig\_number\_10620SRS021484\_contig\_number\_contig-100\_1646.174813SRS021948\_contig\_number\_contig-100\_2594.151984SRS022137\_contig\_number\_contig-100\_204.135483SRS022609\_contig\_number\_52511SRS024009\_contig\_number\_9989SRS024075\_contig\_number\_13131SRS024331\_contig\_number\_contig-100\_2204.205814SRS024549\_contig\_number\_1605SRS024663\_contig\_number\_3391SRS043411\_contig\_number\_15423SRS043841\_contig\_number\_contig-100\_781.80246SRS045004\_contig\_number\_contig-100\_1678.188455SRS046502\_contig\_number\_contig-100\_3578.70742SRS047014\_contig\_number\_contig-100\_11510.191710SRS047433\_contig\_number\_contig-100\_868.133803SRS048870\_contig\_number\_22687SRS049402\_contig\_number\_contig-100\_1487.42643SRS049712\_contig\_number\_contig-100\_4319.112201SRS049959\_contig\_number\_48281SRS049995\_contig\_number\_contig-100\_2347.219413SRS050299\_contig\_number\_contig-100\_2172.32646SRS050422\_contig\_number\_35204SRS050752\_contig\_number\_27909SRS050925\_contig\_number\_37236SRS050941\_contig\_number\_contig-100\_194.138002SRS050998\_contig\_number\_8822SRS051031\_contig\_number\_37689SRS051610\_contig\_number\_10570SRS052697\_contig\_number\_29235SRS053398\_contig\_number\_contig-100\_730.163864SRS055017\_contig\_number\_24768SRS055982\_contig\_number\_19281SRS056259\_contig\_number\_contig-100\_3422.154446SRS056519\_contig\_number\_contig-100\_581.81644SRS058070\_contig\_number\_contig-100\_823.189376SRS063190\_contig\_number\_contig-100\_1456.85833SRS063370\_contig\_number\_3895SRS065176\_contig\_number\_contig-100\_203.108694SRS075078\_contig\_number\_28332SRS075821\_contig\_number\_6483SRS077086\_contig\_number\_9355SRS077552\_contig\_number\_contig-100\_6076.139705SRS078176\_contig\_number\_15999SRS078419\_contig\_number\_37783SRS098571\_contig\_number\_66519SRS098827\_contig\_number\_13603SRS098881\_contig\_number\_23504SRS104084\_contig\_number\_13935SRS1041090\_contig\_number\_1313SRS1041091\_contig\_number\_22585SRS1041116\_contig\_number\_47SRS1041134\_contig\_number\_10988SRS1041146\_contig\_number\_1009SRS104327\_contig\_number\_contig-100\_243.54511SRS104400\_contig\_number\_29624SRS105082\_contig\_number\_contig-100\_368.140115SRS1055049\_contig\_number\_contig-100\_12129.52264SRS1055067\_contig\_number\_14943SRS1055069\_contig\_number\_12162SRS1055076\_contig\_number\_contig-100\_1112.83897SRS1055083\_contig\_number\_9615SRS142542\_contig\_number\_contig-100\_739.166381SRS143372\_contig\_number\_contig-100\_3123.76003SRS143417\_contig\_number\_31408SRS143780\_contig\_number\_13525SRS143876\_contig\_number\_37313SRS143895\_contig\_number\_contig-100\_130.263537SRS144506\_contig\_number\_45229SRS144714\_contig\_number\_contig-100\_7157.141534SRS144753\_contig\_number\_10126SRS145308\_contig\_number\_contig-100\_647.648SRS146812\_contig\_number\_contig-100\_3403.288092SRS148196\_contig\_number\_38371SRS148319\_contig\_number\_7842SRS148424\_contig\_number\_36355SRS148817\_contig\_number\_11927SRS149879\_contig\_number\_26468SRS893270\_contig\_number\_15851SRS893295\_contig\_number\_3383SRS893300\_contig\_number\_contig-100\_3534.67804SRS893341\_contig\_number\_11503
2109687

species
3

SRS022609\_contig\_number\_50559SRS075878\_contig\_number\_22789SRS077231\_contig\_number\_contig-100\_6598.126292
1945593

species
1

SRS893270\_contig\_number\_contig-100\_1768.103584
1263547

1897011

SRS016989\_contig\_number\_17347
1
species

541000
42
family

40
2292270

SRS012849\_contig\_number\_36830SRS014459\_contig\_number\_contig-100\_20.236062SRS014736\_contig\_number\_9428SRS015065\_contig\_number\_34059SRS015431\_contig\_number\_74834SRS017701\_contig\_number\_20686SRS019068\_contig\_number\_91946SRS020394\_contig\_number\_17168SRS022071\_contig\_number\_38424SRS023526\_contig\_number\_25974SRS045826\_contig\_number\_15388SRS053214\_contig\_number\_20575SRS053649\_contig\_number\_contig-100\_4.141233SRS054905\_contig\_number\_contig-100\_19.146471SRS054956\_contig\_number\_1572SRS058770\_contig\_number\_29133SRS063518\_contig\_number\_23969SRS064276\_contig\_number\_38152SRS065397\_contig\_number\_13574SRS075341\_contig\_number\_25852SRS075984\_contig\_number\_27103SRS076976\_contig\_number\_21043SRS077024\_contig\_number\_11661SRS1041095\_contig\_number\_4678SRS1041138\_contig\_number\_14447SRS1055022\_contig\_number\_contig-100\_37.149362SRS1055099\_contig\_number\_13840SRS140492\_contig\_number\_34427SRS144183\_contig\_number\_40815SRS145497\_contig\_number\_20710SRS146813\_contig\_number\_13646SRS147271\_contig\_number\_contig-100\_33.244152SRS148159\_contig\_number\_58050SRS148253\_contig\_number\_contig-100\_259.93113SRS148511\_contig\_number\_contig-100\_9.137405SRS148721\_contig\_number\_55557SRS149784\_contig\_number\_15412SRS893378\_contig\_number\_18509SRS971275\_contig\_number\_46748SRS971276\_contig\_number\_26698
species

2292180

SRS018541\_contig\_number\_8352
1
species

genus
1263
1


SRS893383\_contig\_number\_36113
165186
1
species

species
1897045

SRS011239\_contig\_number\_25191SRS065504\_contig\_number\_38511SRS147919\_contig\_number\_20676
3


SRS1041139\_contig\_number\_6682SRS893373\_contig\_number\_20335
172733
2
species


SRS1041133\_contig\_number\_20727
1897049
1
species

family
186806
3

genus
1730
3

species
39496

SRS046717\_contig\_number\_contig-100\_5396.5397SRS051882\_contig\_number\_contig-100\_2186.126088SRS147139\_contig\_number\_27878
3

family
2
31979

1485
1
genus

species
1

SRS148721\_contig\_number\_54376
59620

580596
1
genus

species
1828555

SRS1055034\_contig\_number\_11889
1

family
1
186803

species

SRS015694\_contig\_number\_10469
39491
1

2109688

SRS011134\_contig\_number\_38775SRS011134\_contig\_number\_49058SRS011302\_contig\_number\_24183SRS012849\_contig\_number\_36882SRS014235\_contig\_number\_43228SRS014459\_contig\_number\_contig-100\_2320.238362SRS014948\_contig\_number\_17402SRS015065\_contig\_number\_contig-100\_3122.208074SRS015431\_contig\_number\_88453SRS015579\_contig\_number\_contig-100\_1682.156193SRS015663\_contig\_number\_34584SRS016203\_contig\_number\_10486SRS016629\_contig\_number\_contig-100\_2584.2585SRS017103\_contig\_number\_contig-100\_131.237827SRS017821\_contig\_number\_15627SRS018313\_contig\_number\_contig-100\_482.75046SRS018351\_contig\_number\_17519SRS018817\_contig\_number\_23660SRS018936\_contig\_number\_contig-100\_995.92804SRS019068\_contig\_number\_65763SRS019267\_contig\_number\_5482SRS019397\_contig\_number\_10547SRS024132\_contig\_number\_44106SRS024132\_contig\_number\_contig-100\_5235.249344SRS024331\_contig\_number\_contig-100\_765.204375SRS024435\_contig\_number\_12012SRS024625\_contig\_number\_contig-100\_968.120783SRS024663\_contig\_number\_15181SRS043001\_contig\_number\_contig-100\_912.62900SRS045645\_contig\_number\_34586SRS045826\_contig\_number\_24198SRS047433\_contig\_number\_contig-100\_1017.133952SRS048164\_contig\_number\_31481SRS049446\_contig\_number\_10982SRS050026\_contig\_number\_9351SRS050941\_contig\_number\_20410SRS052697\_contig\_number\_contig-100\_4305.220910SRS055017\_contig\_number\_36932SRS056273\_contig\_number\_34535SRS057480\_contig\_number\_2250SRS058070\_contig\_number\_contig-100\_127.188680SRS075078\_contig\_number\_contig-100\_2382.183925SRS075341\_contig\_number\_24906SRS075821\_contig\_number\_25091SRS075984\_contig\_number\_13578SRS077024\_contig\_number\_11192SRS077392\_contig\_number\_42601SRS078176\_contig\_number\_23856SRS078242\_contig\_number\_21301SRS078419\_contig\_number\_contig-100\_87.227134SRS078665\_contig\_number\_9111SRS098073\_contig\_number\_13811SRS098514\_contig\_number\_contig-100\_1062.4937SRS098571\_contig\_number\_66475SRS098881\_contig\_number\_17185SRS100907\_contig\_number\_7702SRS101376\_contig\_number\_contig-100\_266.168777SRS1041031\_contig\_number\_29645SRS1041031\_contig\_number\_contig-100\_209.160723SRS1041037\_contig\_number\_6048SRS1041039\_contig\_number\_19673SRS1041137\_contig\_number\_33404SRS1041142\_contig\_number\_contig-100\_2250.2251SRS1041143\_contig\_number\_contig-100\_8796.8797SRS1041157\_contig\_number\_6850SRS104327\_contig\_number\_5954SRS104400\_contig\_number\_12522SRS1055022\_contig\_number\_18155SRS1055038\_contig\_number\_3374SRS1055043\_contig\_number\_12190SRS1055067\_contig\_number\_contig-100\_2262.100861SRS1055099\_contig\_number\_15497SRS140492\_contig\_number\_contig-100\_283.206457SRS142503\_contig\_number\_37224SRS142542\_contig\_number\_20378SRS143070\_contig\_number\_17711SRS143148\_contig\_number\_5991SRS143342\_contig\_number\_20152SRS143417\_contig\_number\_31324SRS143523\_contig\_number\_23611SRS143523\_contig\_number\_26767SRS143876\_contig\_number\_27580SRS144506\_contig\_number\_contig-100\_2341.315063SRS146813\_contig\_number\_contig-100\_2971.165440SRS147271\_contig\_number\_43289SRS148253\_contig\_number\_14319SRS148424\_contig\_number\_contig-100\_1242.197109SRS148784\_contig\_number\_1499SRS148817\_contig\_number\_3716SRS148874\_contig\_number\_5689SRS149325\_contig\_number\_7452SRS893170\_contig\_number\_8523SRS893300\_contig\_number\_6124SRS893366\_contig\_number\_contig-100\_335.155786SRS893383\_contig\_number\_33275SRS971275\_contig\_number\_contig-100\_132.277791
96
species
